# Retraction statement: miR‐944 inhibits metastasis of gastric cancer by preventing the epithelial–mesenchymal transition via MACC1/Met/AKT signaling

**DOI:** 10.1002/2211-5463.13512

**Published:** 2022-11-15

https://doi.org/10.1002/2211-5463.12215

The above article published online on 07 March 2017 in Wiley Online Library (wileyonlinelibrary.com), has been retracted by agreement between the Editor‐in‐Chief, John Wiley and Sons Ltd and the authors. The retraction has been agreed following an investigation based on allegations raised by a third party, which revealed that some images in this article are inappropriate duplications of images from a previously published article [1]. Thus, the editors consider the conclusions of this manuscript substantially compromised.
